# Feasibility of Using a Mobile App Supported Executive Function Intervention in Military Service Members and Veterans with mTBI and Co-Occurring Psychological Conditions

**DOI:** 10.3390/ijerph20032457

**Published:** 2023-01-30

**Authors:** Rebecca Gartell, John Morris, Tracey Wallace

**Affiliations:** 1 SHARE Military Initiative, Shepherd Center, Atlanta, GA 30309, USA; 2 Virginia C. Crawford Research Institute, Shepherd Center, Atlanta, GA 30309, USA

**Keywords:** brain injuries, concussion, military personnel, executive function, problem solving, emotional regulation, rehabilitation, technology, smartphone, telehealth

## Abstract

This pilot study assessed the feasibility of using SwapMyMood, a smartphone application supporting evidence-based strategies for emotion regulation and problem-solving as a supplement to conventional care for military service members and veterans (SM/Vs) experiencing chronic symptoms of mild traumatic brain injury (mTBI) and co-occurring psychological conditions. Eight military SM/Vs were recruited from an intensive outpatient program. Participants were block randomized to an experimental group (conventional care plus use of the SwapMyMood app) or a conventional care only group for six weeks. Conventional care included instruction on problem-solving and emotion regulation strategies using traditional paper manuals and protocols. Effects on the knowledge and use of strategies and related goal attainment were measured. Patient-reported outcomes were measured via several validated problem-solving and emotion regulation scales. No differences were found between groups in goal attainment, global executive function, problem-solving, emotion regulation, and knowledge of how to use the problem-solving and emotion regulation strategies targeted. Experimental group participants rated the application positively, demonstrating feasibility of integration of the app into clinical care. The implementation of SwapMyMood is feasible in a clinical setting. SwapMyMood may be a clinically effective supplemental tool for supporting executive function in SM/Vs with mTBI and co-occurring psychological conditions.

## 1. Introduction

Between 2000 and 2022, 458,894 military service members were diagnosed with traumatic brain injury (TBI), with the majority being mild TBI (mTBI) [1]. Most individuals who sustain an mTBI improve within several days or weeks following injury. However, at least 20% experience cognitive, physical, emotional, or sleep symptoms lasting longer [2,3,4,5,6]. Commonly reported changes in cognitive function following mTBI include alterations in memory, attention, and executive functioning. Chronic cognitive complaints following mTBI are associated with conditions such as chronic pain, sleep disorders, and emotional changes including increased anxiety and depression. [7,8]. Chronic symptoms may negatively affect community integration, vocational performance, interpersonal relationships, and academic achievement as well as work synergistically with symptoms of other psychological health conditions (e.g., post-traumatic stress disorder (PTSD)) that can co-occur in military service members and veterans (SM/Vs) [9]. The acronyms and abbreviations used in this article are listed in Table 1.

Several studies support the use of interventions that concurrently address cognition and co-occurring psychological conditions following mTBI, including the use of metacognitive strategy instruction (thinking about one’s thinking) [10,11,12,13,14]. The Executive Plus/STEP program incorporates metacognitive strategy training to support executive functioning, namely problem-solving (SWAPS strategy) and emotion regulation (Emotional Cycle strategy) for individuals who have sustained a brain injury. The program has been shown to be efficacious in improving self-reported post-TBI executive function and problem solving, helping individuals identify and implement solutions to problems encountered in daily life and regulate their emotional responses to these challenges [15]. Preliminary results of a group-based videoconference format of the program also have shown improvement in emotion regulation in individuals who have a history of TBI [16].

Barriers to the successful use of interventions by individuals with TBI include lack of access to the recommended frequency of treatment and challenges applying interventional tools to environments outside of supportive treatment settings [17,18,19]. In addition, intervention models often focus on the implementation of paper/pencil-based manuals and protocols which have limits on transportability and accessibility. When implementing the Executive Plus/STEP program with military SM/Vs with a history of mTBI and co-occurring psychological conditions, the authors have observed that some report difficulty recalling multiple steps in interventions, recalling successes, and using strategies under stress. SwapMyMood is a mobile app version of the established and successful Executive Plus/STEP program designed to address these barriers [15,20]. It provides a portable, user-friendly electronic version of Executive Plus/STEP tools previously available in paper format only. The app was developed by researchers and clinicians at Shepherd Center, in Atlanta, Georgia, USA and published by Shepherd Center, Inc. Development and testing of SwapMyMood was supported by the Rehabilitation Engineering Research Center for Community Living, Health and Function (LiveWell RERC) funded by a grant from the National Institute on Disability, Independent Living, and Rehabilitation Research (NIDILRR).

**Figure 1:**
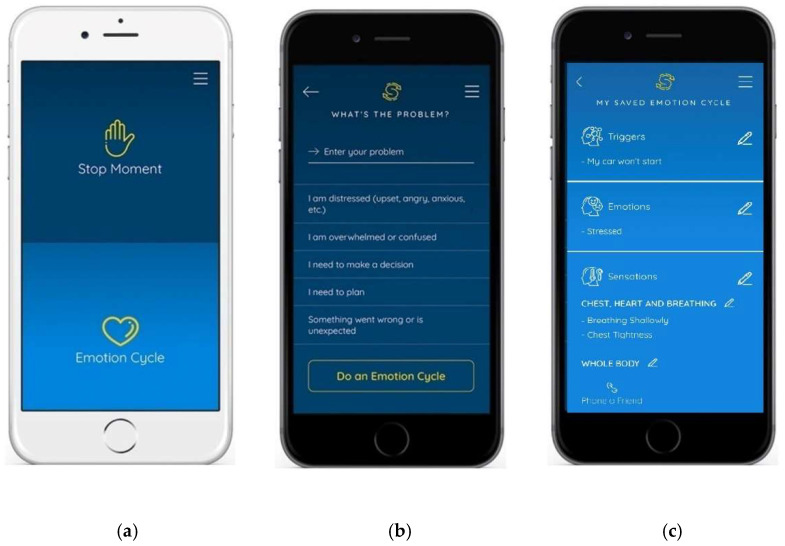
SwapMyMood app: (**a**) The home screen provides user with options to begin the SWAPS problem-solving strategy by entering a Stop Moment or to begin an Emotional Cycle. (**b**) The “What’s the Problem” screen guides the user to complete a step in the SWAPS problem-solving strategy by identifying the problem, and it also provides the option for the user to incorporate completion of an Emotional Cycle to help identify all parts of the problem, including how it is affecting them. (**c**) The “My Saved Emotion Cycle” screen displays user identified aspects of the Emotional Cycle strategy in an editable format.

SwapMyMood addresses barriers to traditional metacognitive strategy instruction by offering personalized solutions to individualized challenges that have shown to be effective for real-time situational coping. It supports users in recalling and implementing multi-step, executive functioning strategies. The app also provides feedback about actions that have previously been successful in helping the user with problem-solving and emotion regulation (Figure 1).

The research questions for this feasibility study are: (1) will the addition of the use of SwapMyMood result in at least equivalent clinically meaningful change in self-reported executive functioning and goal attainment compared to use of Executive Plus/STEP Program paper manuals alone in SM/Vs with mTBI and co-occurring psychological conditions?; (2) will SM/Vs with mTBI and co-occurring psychological conditions rate SwapMyMood usability as acceptable?; and (3) is the study design feasible for conducting this research within a clinical setting? The research protocol was embedded in a clinical program to provide insight on intervention implementation in a real-world healthcare setting. 

## 2. Materials and Methods

The study protocol was approved for use of human subjects by the Shepherd Center Institutional Review Board. Clinicians were directly involved in the study design, administration, and data collection. Participants, intervention providers, and those assessing the outcomes were not blinded.

Feasibility studies are usually conducted in preparation for a larger, main study [21]. They can evaluate components of an intervention and its implementation as well as components of the study design (e.g., sampling, effect size, etc.), and they can include a range of aims and methods. Arain et al. [22] list several possible specific aims of feasibility studies, including identifying: (1) the standard deviation of outcome measures, which may be needed for estimating sample size, (2) willingness of participants to participate and be randomized, (3) willingness and ability of clinicians to recruit participants, (4) number of patients who fit inclusion criteria, (5) characteristics of the proposed outcome measures, (6) follow-up rates, response rates to questionnaires, and adherence/compliance rates.

This feasibility study focuses on three core aims. The first aim, evaluating preliminary efficacy of the intervention, was addressed by measuring participant goal attainment, change in clinical measures of executive function, and change in self-assessment of knowledge of how to use the intervention. The outcomes of participants who received the SwapMyMood app were compared to outcomes of those who only received conventional care. The second aim, measuring acceptability of the SwapMyMood app by target users, was measured by using a standardized usability scale and gathering feedback from participants regarding their experience using the app. The third aim, assessing the study design for implementation in a target clinical setting, was achieved by evaluating the quality and completeness of data collected as well as the ease/difficulty of recruiting and retaining participants within the context of an outpatient program serving SM/Vs with mTBI and co-occurring psychological conditions.

### 2.1. Intervention

SwapMyMood was developed following principles of user-centered design, engaging stakeholders at each stage of development as previously described [23]. Subject matter experts and target users provided feedback throughout the process. A proof of concept was created and then updated and assessed for usability by 12 subject matter experts and 16 target users via interviews, sit-by demonstrations, and take-home testing [20]. Modifications to the proof of concept were made following initial target user and subject matter expert feedback, resolving technical issues and resulting in a fully integrated functional mobile app available for both iOS and Android. These modifications included a new, more robust version of the app, a streamlined design and layout, additional images and graphical elements, more user account features, and the creation of a clinician administration (admin) portal that allows app administrators to view app use data (e.g., frequency of use, types of problems encountered) for app users who grant permission.

### 2.2. Participants and Procedures

A total of 8 participants were recruited from the SHARE Military Initiative (SHARE) intensive outpatient program at Shepherd Center in Atlanta, Georgia. SHARE is a donor-funded program that provides comprehensive, intensive interdisciplinary outpatient rehabilitation care to military SM/Vs experiencing symptoms of brain injury and co-occurring psychological conditions in a civilian setting. Trained study staff approached each eligible SHARE client to inquire about participation in the current study. Prospective participants were assured that their decision to participate or not in the current study would have no bearing on their clinical care. Clients expressing interest were scheduled for a subsequent research visit, and informed consent was obtained. Participants were enrolled in the study and received care at different times based on when they were admitted to SHARE. 

Inclusion criteria:

- Diagnosis of mTBI at least 6 months post-onset;
- Identified by SHARE behavioral health (BH) provider or speech-language pathologist (SLP) as appropriate for Executive Plus/STEP interventions;
- 18 years of age or older;
- Functional hearing and vision;
- Able to follow 2-step directions;
- English fluency;
- Functional reading;
- iOS or Android smartphone user; and
- Speaks or types functionally for smartphone use.

Exclusion criteria:

- Participation in SHARE for less than 6 weeks;
- Other conditions that may impact cognitive functioning (e.g., stroke, psychosis).

Prior to study enrollment, all participants received instruction in use of the Executive Plus/STEP strategies from experienced SLP and BH providers in group therapy sessions as part of standard care. Continued training in the use of Executive Plus/STEP strategies was administered via weekly SLP group sessions and BH group sessions, each lasting 60 minutes. Homework assignments, individualized to the participant and determined through patient–provider collaboration, were given to support use outside of the clinical setting. Data on specifics and frequency of homework were not included in this study. Due to the COVID-19 pandemic, some participants received treatment via telehealth during portions of the program. 

Eligible participants who provided consent were block randomized to one of two groups. Conventional care group participants received ongoing Executive Plus/STEP strategy training using traditional paper manuals and protocols without the use of any mobile app, while experimental group participants received the same training and were provided version 1.0.4 of the SwapMyMood app, which was downloaded to their personal smartphone. In order to prevent cross-contamination between the conventional care group and the experimental group who were all receiving treatment in the same clinical setting, we block randomized such that 50% of all the participants were assigned to the conventional care group first. We then assigned the remaining 50% of participants to the experimental group.

### 2.3. Measures

Participants completed assessments pre- and post-intervention. Pre-measures were recorded following a 2-week evaluation that was completed as part of the clinical program; post-measures were completed following a 6-week treatment period (Table 2). Measures for this study were collected in-person or via a telehealth session. Assessments took approximately one hour to complete and were conducted one week prior and within one week subsequent to the 6-week treatment period.

Goal Attainment Scaling (GAS), an individualized outcome measure involving goal selection and scaling, and standardized to calculate level of goal attainment over the course of an intervention, was used as the primary outcome measure [24]. GAS has been shown to have satisfactory to excellent psychometric properties [24] and has been shown to be effective for measuring attainment of person-centered goals of military SM/Vs with mTBI [25]. Goals were individually identified to suit the participant by targeting improvement in problem-solving and/or emotion regulation during personally relevant functional tasks, and the scales were personalized around their current and expected levels of performance, thus supporting the measurement of meaningful change in executive function in the context of each participant’s life.

Participants also completed pre–post standardized, self-report assessments of executive functioning. Overall executive functioning was measured via the Behavior Rating Inventory of Executive Function-Adult Version (BRIEF-A) [26], confidence and performance in problem-solving was measured via the Problem-Solving Inventory (PSI) [27], and emotion regulation was measured via the Difficulties in Emotion Regulation Scale (DERS) [28]. The BRIEF-A was selected because its item-level psychometrics have been demonstrated as adequate in TBI populations [29], it has been used previously to measure everyday executive function behaviors in military mTBI intervention studies [30], and the measure was already being administered as part of the clinical program at SHARE, thereby reducing the participant burden related to completion of both clinical and study measures. The PSI and DERS were selected because they were used in prior work evaluating changes in problem-solving and emotion regulation in people with TBI following the implementation of study interventions [15]. In addition, a four-point Likert scale was used to capture participant self-reported knowledge of how to use the Executive Plus/STEP strategies by asking, “How well do you know how to use SWAPS to support problem-solving and planning?”, and “How well do you know how to use the Emotional Cycle to support emotion regulation?”. Experimental group participants also completed the System Usability Scale (SUS), which is a self-report measure of technology product usability [31] and answered a series of questions regarding their experience using the app.

### 2.4. Data Analysis

Participant characteristics, such as age, race, and gender, are presented at an individual-patient level. Outcomes are described using counts/percentages for categorical variables and means/standard deviations for scale variables. While under-powered, initial comparisons between groups were conducted using the χ2/exact test for categorical variables and Mann–Whitney U test for scale variables. In order to conduct adjusted comparisons, propensity scores were first computed using age, race, gender, type of sessions (e.g., hybrid, in person, etc.), hours of group treatment addressing the interventions, time since injury, education, diagnosis of PTSD, and score at admission. Due to the sample size, a ridge penalty was used to compute propensity scores. Comparisons were then repeated using the Cochran–Mantel–Haenszel test and Quade rank-order analysis of covariance for categorical and scale outcomes, respectively. Analyses were conducted using R (v4.2) [32].

## 3. Results

Eight participants were enrolled, with four participants assigned to each group. No difficulties recruiting or retaining participants were identified by the study staff. Overall, participants were well educated with most completing at least some college, although conventional care group participants were more educated. Ages ranged from 22 to 54 (M = 37.4), with little diversity in race or service branch, as most were non-Hispanic White (*n* = 7) and were army SM/Vs (*n* = 6). Gender identity included female (*n* = 2), male (*n* = 5), and non-binary (*n* = 1) participants via self-report (Table 3).

Participant injury and treatment characteristics are summarized in Table 4. The number of diagnosed mTBIs was well matched between groups, as was primary mechanism of injury (blast exposure; *n* = 6), with experimental group participants more frequently experiencing multiple mechanisms of injury. Primary psychological health diagnoses were PTSD (*n* = 4) and Major Depressive Disorder (*n* = 4). Years post-onset of index mTBI ranged from 0.5–15 (M = 5.4). Service delivery method was evenly distributed, although conventional care participants received more group therapy sessions (M = 9.5) than experimental group participants (M = 7.5).

Table 5 describes overall and between group change for outcome measures. Groups demonstrated improvement on and were statistically comparable on all outcome measures. All participants met or exceeded their goal, receiving a score of 0 or greater on the GAS at study completion and thereby demonstrating meaningful improvement. Experimental group participants rated the app highly on the SUS (M = 77.5; range = 57.5–92.5), which uses a scale ranging from 0 to 100 where 100 is the highest usability rating possible. The four participants indicated they either agreed or strongly agreed that they felt very confident using the app, and they would imagine that most people would learn to use SwapMyMood very quickly. Regarding their experience using the app, three of the four experimental group participants reported the app was “very easy” to use, and two participants stated they preferred the app over the paper manuals.

## 4. Discussion

Results of this pilot feasibility study suggest SwapMyMood may be an acceptable addition to the use of paper manuals to support SM/Vs with mTBI and co-occurring psychological conditions in using the executive functioning strategies taught in the Executive Plus/STEP Program and should be studied in an adequately powered, randomized controlled trial. The groups were statistically comparable on all outcome measures, both with and without adjustments for group difference in demographic, injury, and treatment characteristics, and both groups demonstrated post-intervention gains in goal attainment, executive function, problem-solving, emotion regulation, and knowledge of how to use the strategies targeted. Furthermore, all participants in both groups demonstrated meaningful change in executive function as measured by GAS. The present study does not control for or consider the impact of other rehabilitation services and interventions participants received during the study period, and thus, gains in executive function cannot be interpreted as resultant of the study intervention. However, the equivalent gains demonstrated by the groups provide preliminary support for use of SwapMyMoood as an adjunct option to conventional care, adding evidence for the feasibility of use of computerized technology for cognitive and behavioral interventions for people with brain injury [33,34,35]. Notably, participants were randomized to groups rather than matched to the intervention option (i.e., the paper manual or app) according to their preferences and abilities [36] as is appropriate for clinician implementation outside of research. Therefore, it is unknown whether matching persons to intervention options would yield different results. In addition, experimental group app users rated the SwapMyMood highly for usability. While two of the four experimental group app users reported a preference for the app over the paper manuals, the impact of individual participant attitudes toward and preferences for technology in general is not known nor is it known whether they adopted the technology long term. Furthermore, no difficulties in recruiting or retaining participants were identified, and the demonstrated feasibility of the assessment and clinical protocol used in this study is also informative for future investigations.

These findings suggest feasibility by demonstrating effective implementation of the SwapMyMood app in a clinical setting in this sample. This intervention was implemented during the COVID-19 pandemic when in-person clinical interventions were not available for every client. This study suggests that SwapMyMood may be an acceptable intervention which can be utilized for remote use for people with brain injury who are unable to obtain in-person care (e.g., due to financial or transportation limitations or disruptions such those caused by the COVID-19 pandemic). Limited access to rehabilitation services after TBI for individuals in underserved groups is well documented in the literature [37,38]. The convenience of an easy to use, free electronic application to help appropriate individuals utilize tools for problem-solving and emotion regulation following TBI could potentially mitigate some barriers to healthcare access. Due to the small sample size, generalizability is limited. These findings provide support for conducting future research with a larger sample.

### 4.1. Limitations

The primary limitation of this study is the small sample size, which limits statistical analysis and the generalizability of findings. While both groups demonstrated post-intervention gains and appear statistically comparable, it is unknown whether differences may be detectable in a larger sample. While statistically non-significant, it is notable that the conventional care group received more hours of group instruction compared to the experimental group, who had more frequent absences and scheduling conflicts. It is unknown whether this negatively impacted experimental group outcomes. Additional training on the interventions during other treatment sessions at SHARE or elsewhere was not tracked nor was the completion of homework and frequency of use of the interventions outside of group sessions. Therefore, group differences and the potential impact of those differences is unknown. The impact of the psychological conditions (Table 4) on intervention outcomes is also unknown. Although the Executive Plus/STEP protocol has previously been shown to be a beneficial intervention for self-reported measure of executive function and problem-solving challenges in individuals with brain injury [15], its effectiveness has not been previously researched in a population of SM/Vs with co-occurring psychological conditions.

This study was conducted in a real-world clinical setting during the COVID-19 global pandemic, which required some participants to receive a hybrid program including interventions delivered via a telehealth platform in addition to in-person care. While half of the participants in each group received hybrid care, the exact number of in-person versus virtual group therapy sessions was not considered in the analysis. Furthermore, the efficacy of telehealth delivery of the Executive Plus/STEP protocol in comparison to in-person care has not been robustly studied, and therefore, the extent to which varied service delivery methods influenced outcomes is unknown.

### 4.2. Future Directions

A central aim of this study was to assess whether SM/Vs with co-occurring psychological conditions would find SwapMyMood acceptable and useful. While most experimental group participants rated the app highly on the SUS, one participant did not. Participant feedback will be considered in refinements to the current version of the SwapMyMood app, which is aimed at improving usability and enhancing design features.

These data will also be used to inform version 3.0 development, to incorporate machine learning and artificial intelligence to support context awareness and predictive modeling to anticipate user needs to utilize problem-solving and emotion regulation tools. These features and functions will enhance support for users by delivering timely prompts, reminders and engagement supports, while minimizing risk of over-communication with the user. Additional enhancements will offer opportunity for clinical providers outside of the research team to access user data in the admin portal to support remote monitoring, care provision, and independent research studies. 

Future research will be conducted in larger controlled trials with a more racially, ethnically, and demographically diverse cohort to ensure replicability of present results.

## 5. Conclusions

SwapMyMood may be a clinically effective supplemental tool for supporting executive function in SM/Vs with mTBI and co-occurring psychological conditions and should be studied in a larger, controlled trial with a more diverse cohort, using methodology similar to that used in this feasibility study.

## Figures and Tables

**Table 1:** Abbreviations.

Abbreviations	Meaning
admin	administration
BRIEF-A	Behavioral Rating Inventory of Executive Function—Adult Version
DERS	Difficulties in Emotion Regulation Scale
GAS	goal attainment scaling
mTBI	mild traumatic brain injury
PSI	Problem-Solving Inventory
PTSD	post-traumatic stress disorder
SM/Vs	service members and Veterans
SUS	System Usability Scale

**Table 2:** Outcome Measures.

Assessment	Objective/Description	Scoring/Assessment Type
Goal Attainment Scaling (GAS)	Individualized outcome measure involving goal selection and scaling, standardized to calculate level of goal attainment over the course of an intervention. Goals were individually identified to suit the participant.	5-point scale ranging from −2 to +2 set according to participant’s current and expected levels of performance; a score of 0 or greater indicates meaningful improvement on repeated measures
Behavioral Rating Inventory of Executive Function-Adult Version (BRIEF-A)	Assess executive function	75-item self-report questionnaire with three response options: “Never”, “Sometimes”, “Often”; decreased scores indicate improvement on repeated measures
Problem-Solving Inventory (PSI)	Assess confidence in problem-solving, whether individuals tend to approach or avoid problems, and elements of self-control	32-item self-report using a 6-point rating scale from “Strongly Agree” to “Strongly Disagree”; decreased scores indicate improvement on repeated measures
Difficulties in Emotion Regulation Scale (DERS)	Assess emotion regulation	36-item self-report questionnaire with five response options from “Almost Never” to “Almost Always”; decreased scores indicate improvement on repeated measures
Likert scale	Assess participant knowledge of how to use the Executive Plus/STEP system	Informal 8-item self-report inventory with varied response options; increased scores indicate improvement on repeated measures
System Usability Scale (SUS)	Evaluate usability of technology products and services	10-item questionnaire with five response options from “Strongly Agree” to “Strongly Disagree”; higher scores indicate greater usability

**Table 3:** Participant Demographics.

Group (Participant Number)	Age	Gender	Race	Branch	Highest Level of Education	Personal Phone
CCG (1)	37	Non-binary	White	Army	Graduate degree	Android
CCG (2)	35	Female	White	Navy	Graduate degree	iOS
CCG (3)	22	Male	White	Army	Some college	Android
CCG (4)	45	Male	White	Army	Some college	iOS
EG (5)	35	Male	White	Army	High school	iOS
EG (6)	38	Female	Hispanic	Marines	Some college	iOS
EG (7)	54	Male	White	Army	College	iOS
EG (8)	33	Male	White	Army	College	iOS

Abbreviation: CCG, Conventional Care Group; EG, Experimental Group.

**Table 4:** Participant Injury and Treatment Characteristics.

Group (Participant Number)	Hours of Group Treatment	Treatment Delivery	Years Post Index mTBI	Number of mTBI ^a^	Mechanism of Brain Injury	Psychological Conditions
CCG (1)	9	Hybrid ^b^	5	3+	Blast	AUD, MDD, UAD
CCG (2)	8	In-person	2.5	1	Fall	AUD, UAD, UMD
CCG (3)	9	Hybrid	0.5	1	MVA	PDD, PTSD, UAD
CCG (4)	12	In-person	5	2	BA, Blast	UDD
EG (5)	10	In-person	3	2	SBO, Blast	MDD, PTSD
EG (6)	8	Hybrid	9	1	Sports	MDD, PTSD
EG (7)	6	Hybrid	15	1	Blast	ADU
EG (8)	6	In-person	3	3+	Blast, MVA, SBO	MDD, PTSD

^a^ Number of mTBI includes mTBI diagnosed by provider. For two participants, this includes at least 3 confirmed with more suspected by clinician (denoted above by 3+). ^b^ Hybrid treatment delivery refers to care provided via a combination of in-person and telehealth sessions. Abbreviation: ADU, Adjustment Disorder Unspecified; AUD. Alcohol Use Disorder; BA, Bicycle Accident; CCG, Conventional Care Group; EG, Experimental Group; MDD, Major Depressive Disorder; MVA, Motor Vehicle Accident; PTSD, Post-Traumatic Stress Disorder; SBO, Struck by Object; UAD, Unspecified Anxiety Disorder; UDD, Unspecified Depressive Disorder; UMD, Unspecified Mood Disorder; PDD, Persistent Depressive Disorder.

**Table 5:** Differences Between Groups for Outcome Measures.

	Overall ^a^	CCG ^a^	EG ^a^	Analysis	
	*n* = 8	*n* = 4	*n* = 4	*p*	*p*-adj
Goal Attainment (GAS)					
Post				1	1
0	2 (25)	1 (25)	1 (25)		
1	4 (50)	2 (50)	2 (50)		
2	2 (25)	1 (25)	1 (25)		
BRIEF-A (GEC)					
Pre	78.25 (13.12)	80.75 (8.96)	75.75 (17.46)	–	–
Post	62.75 (16.03)	60.75 (14.29)	64.75 (19.62)	0.69	0.81
Change	−15.50 (14.11)	−20.00 (17.36)	−11.00 (10.46)	0.69	0.94
BRIEF-A (BRI)					
Pre	75.75 (11.51)	76.00 (9.06)	75.50 (15.07)	–	–
Post	59.13 (14.15)	56.75 (16.09)	61.50 (13.89)	0.49	0.88
Change	−16.63 (12.93)	−19.25 (15.46)	−14.00 (11.52)	1	0.81
		BRIEF-A (MCI)			
Pre	75.88 (13.58)	79.75 (8.26)	72.00 (17.94)	–	–
Post	64.25 (16.16)	63.00 (11.92)	65.50 (21.52)	1	0.91
Change	−11.62 (13.16)	−16.75 (15.63)	−6.50 (9.47)	0.48	0.78
PSI					
Pre	116.88 (12.11)	117.25 (14.57)	116.50 (11.39)	–	–
Post	104.50 (16.25)	102.50 (16.60)	106.50 (18.16)	1	0.56
Change	−12.37 (19.63)	−14.75 (13.94)	−10.00 (26.27)	0.89	0.53
DERS					
Pre	129.88 (33.43)	131.00 (37.07)	128.75 (35.08)	–	–
Post	81.13 (28.92)	79.50 (31.84)	82.75 (30.51)	0.69	0.98
Change	−48.75 (51.15)	−51.50 (49.16)	−46.00 (60.55)	1	0.77
Know how to use SWAPS					
Pre	2.38 (0.74)	2.25 (0.50)	2.50 (1.00)	–	–
Post	3.50 (0.54)	3.50 (0.58)	3.50 (0.58)	1	0.84
Change	1.13 (1.13)	1.25 (0.96)	1.00 (1.41)	0.89	0.96
Know how to use Emotional Cycle					
Pre	1.75 (1.04)	2.00 (1.16)	1.50 (1.00)	–	–
Post	3.63 (0.52)	3.50 (0.58)	3.75 (0.50)	0.69	0.98
Change	1.88 (1.13)	1.50 (1.29)	2.25 (0.96)	0.49	0.88

^a^ Data in these columns are presented as mean (SD), with exception to the Goal Attainment data, which are presented as n (%). Abbreviation: BRIEF-A, Behavioral Rating Inventory of Executive Functioning—Adult Version; BRI, Behavioral Regulation Index; CCG, Conventional Care Group; DERS, Difficulties in Emotion Regulation Scale Dysexecutive; EG, Experimental Group; GAS, Goal Attainment Scaling; GEC, Global Executive Composite; MCI, Metacognition Index; *p*, *p* value computed from the χ2/exact test or Mann–Whitney U test; *p*-adj, adjusted *p* value computed from the Cochran–Mantel–Haenszel test or Quade ANCOVA; PSI, Problem-Solving Inventory Note: improvement is indicated by increased scores on goal attainment scaling, knowledge of how to use SWAPS, and knowledge of how to use the Emotion Cycle, and by decreased scores on BRIEF, PSI, and DERS.

## Data Availability

The raw data supporting the conclusions of this article will be made available by the authors without undue reservation.

## References

[1] DOD TBI Worldwide Numbers. https://health.mil/Military-Health-Topics/Centers-of-Excellence/Traumatic-Brain-Injury-Center-of-Excellence/DOD-TBI-Worldwide-Numbers.

[2] Leddy J.J., Sandhu H., Sodhi V., Baker J.G., Willer B. (2012). Rehabilitation of Concussion and Post-Concussion Syndrome. Sport. Health.

[3] Quinn D.K., Mayer A.R., Master C.L., Fann J.R. (2018). Prolonged Postconcussive Symptoms. Am. J. Psychiatry.

[4] McInnes K., Friesen C.L., MacKenzie D.E., Westwood D.A., Boe S.G. (2017). Mild Traumatic Brain Injury (MTBI) and Chronic Cognitive Impairment: A Scoping Review. PLoS ONE.

[5] Madhok D.Y., Rodriguez R.M., Barber J., Temkin N.R., Markowitz A.J., Kreitzer N., Manley G.T. (2022). TRACK-TBI Investigators Outcomes in Patients with Mild Traumatic Brain Injury Without Acute Intracranial Traumatic Injury. JAMA Netw. Open.

[6] Schneider A.L.C., Huie J.R., Boscardin W.J., Nelson L., Barber J.K., Yaffe K., Diaz-Arrastia R., Ferguson A.R., Kramer J., Jain S. (2022). Cognitive Outcome 1 Year After Mild Traumatic Brain Injury: Results From the TRACK-TBI Study. Neurology.

[7] McMahon P., Hricik A., Yue J.K., Puccio A.M., Inoue T., Lingsma H.F., Beers S.R., Gordon W.A., Valadka A.B., Manley G.T. (2014). Symptomatology and Functional Outcome in Mild Traumatic Brain Injury: Results from the Prospective TRACK-TBI Study. J. Neurotrauma.

[8] Izzy S., Tahir Z., Grashow R., Cote D.J., Jarrah A.A., Dhand A., Taylor H., Whalen M., Nathan D.M., Miller K.K. (2021). Concussion and Risk of Chronic Medical and Behavioral Health Comorbidities. J. Neurotrauma..

[9] Cooper D.B., Kennedy J.E., Cullen M.A., Critchfield E., Amador R.R., Bowles A.O. (2011). Association between Combat Stress and Post-Concussive Symptom Reporting in OEF/OIF Service Members with Mild Traumatic Brain Injuries. Brain Inj..

[10] Cicerone K.D., Goldin Y., Ganci K., Rosenbaum A., Wethe J.V., Langenbahn D.M., Malec J.F., Bergquist T.F., Kingsley K., Nagele D. (2019). Evidence-Based Cognitive Rehabilitation: Systematic Review of the Literature from 2009 Through 2014. Arch. Phys. Med. Rehabil..

[11] Tate R., Kennedy M., Ponsford J., Douglas J., Velikonja D., Bayley M., Stergiou-Kita M. (2014). INCOG Recommendations for Management of Cognition Following Traumatic Brain Injury, Part III: Executive Function and Self-Awareness. J. Head Trauma Rehabil..

[12] Shoulson I., Wilhelm E.E., Koehler R. (2013). Cognitive Rehabilitation Therapy for Traumatic Brain Injury: Evaluating the Evidence. FOC.

[13] Eapen B.C., Bowles A.O., Sall J., Lang A.E., Hoppes C.W., Stout K.C., Kretzmer T., Cifu D.X. (2022). The Management and Rehabilitation of Post-Acute Mild Traumatic Brain Injury. Brain Inj..

[14] Rytter H.M., Westenbaek K., Henriksen H., Christiansen P., Humle F. (2019). Specialized Interdisciplinary Rehabilitation Reduces Persistent Post-Concussive Symptoms: A Randomized Clinical Trial. Brain Inj..

[15] Cantor J., Ashman T., Dams-O’Connor K., Dijkers M.P., Gordon W., Spielman L., Tsaousides T., Allen H., Nguyen M., Oswald J. (2014). Evaluation of the Short-Term Executive plus Intervention for Executive Dysfunction after Traumatic Brain Injury: A Randomized Controlled Trial with Minimization. Arch. Phys. Med. Rehabil..

[16] Tsaousides T., Spielman L., Kajankova M., Guetta G., Gordon W., Dams-O’Connor K. (2017). Improving Emotion Regulation Following Web-Based Group Intervention for Individuals with Traumatic Brain Injury. J. Head Trauma Rehabil..

[17] Wong D., Sinclair K., Seabrook E., McKay A., Ponsford J. (2017). Smartphones as Assistive Technology Following Traumatic Brain Injury: A Preliminary Study of What Helps and What Hinders. Disabil. Rehabil..

[18] de Joode E., van Heugten C., Verhey F., van Boxtel M. (2010). Efficacy and Usability of Assistive Technology for Patients with Cognitive Deficits: A Systematic Review. Clin. Rehabil..

[19] LoPresti E.F., Bodine C., Lewis C. (2008). Assistive Technology for Cognition. IEEE Eng. Med. Biol. Mag..

[20] Wallace T.D., Morris J.T., Miesenberger K., Manduchi R., Covarrubias Rodriguez M., Peňáz P. (2020). SwapMyMood: User-Centered Design and Development of a Mobile App to Support Executive Function. Computers Helping People with Special Needs.

[21] Pearson N., Naylor P.J., Ashe M.C., Fernandez M., Yoong S.L., Wolfenden L. (2020). Guidance for conducting feasibility and pilot studies for implementation trials. Pilot Feasibility Stud..

[22] Arain M., Campbell M.J., Cooper C.L., Lancaster G.A. (2010). What is a pilot or feasibility study? A review of current practice and editorial policy. BMC Med. Res. Methodol..

[23] Luna D., Quispe M., Gonzalez Z., Alemrares A., Risk M., Garcia Aurelio M., Otero C. (2015). User-Centered Design to Develop Clinical Applications. Literature Review. Stud. Health Technol. Inform..

[24] Malec J.F. (1999). Goal Attainment Scaling in Rehabilitation. Neuropsychol. Rehabil..

[25] Wallace T.D., McCauley K.L., Hodge A.T., Moran T.P., Porter S.T., Whaley M.C., Gore R.K. (2022). Use of person-centered goals to direct interdisciplinary care for military service members and Veterans with chronic mTBI and co-occurring psychological conditions. Front. Neurol..

[26] Roth R.M., Isquith P.K., Gioia G.A. (2005). Behavior Rating Inventory of Executive Function-Adult Version (BRIEF-A).

[27] Heppner P.P., Petersen C.H. (1982). The Development and Implications of a Personal Problem-Solving Inventory. J. Couns. Psychol..

[28] Gratz K.L., Roemer L. (2004). Multidimensional Assessment of Emotion Regulation and Dysregulation: Development, Factor Structure, and Initial Validation of the Difficulties in Emotion Regulation Scale. J. Psychopathol. Behav. Assess..

[29] Waid-Ebbs J.K., Wen P.S., Heaton S.C., Donovan N.J., Velozo C. (2012). The item level psychometrics of the behaviour rating inventory of executive function-adult (BRIEF-A) in a TBI sample. Brain Inj..

[30] Bergersen K., Halvorsen J.Ø., Tryti E.A., Taylor S.I., Olsen A. (2017). A systematic literature review of psychotherapeutic treatment of prolonged symptoms after mild traumatic brain injury. Brain Inj..

[31] Brooke J. (1996). SUS: A “Quick and Dirty” Usability Scale. Usability Evaluation in Industry.

[32] R Core Team (2022). R: A Language and Environment for Statistical Computing.

[33] Powell L.E., Wild M.R., Glang A., Ibarra S., Gau J.M., Perez A., Albin R.W., O’Neil Pirozzi T.M., Wade S.L., Keating T. (2019). The development and evaluation of a web-based programme to support problem-solving skills following brain injury. Disabil. Rehabil. Assist. Technololgy..

[34] Rabinowitz A.R., Collier G., Vaccaro M., Wingfield R. (2022). Development of RehaBot—A Conversational Agent for Promoting Rewarding Activities in Users with Traumatic Brain Injury. J. Head Trauma Rehab.

[35] Wallace T., Morris J.T., Glickstein R., Anderson R.K., Gore R.K. (2002). Implementation of a Mobile Technology-Supported Diaphragmatic Breathing Intervention in Military mTBI with PTSD. J. Head Trauma Rehab.

[36] Scherer M.J., Federici S. (2015). Why people use and don’t use technologies: Introduction to the special issue on assistive technologies for cognition/cognitive support technologies. NeuroRehabilitation.

[37] Schiraldi M., Patil C.G., Mukherjee D., Ugiliweneza B., Nuño M., Lad S.P., Boakye M. (2015). Effect of Insurance and Racial Disparities on Outcomes in Traumatic Brain Injury. J. Neurol. Surg. A Cent. Eur. Neurosurg..

[38] Arango-Lasprilla J.C., Ketchum J.M., Gary K., Hart T., Corrigan J., Forster L., Mascialino G. (2009). Race/Ethnicity Differences in Satisfaction with Life among Persons with Traumatic Brain Injury. NeuroRehabilitation.

